# Flavonoids and the CNS

**DOI:** 10.3390/molecules16021471

**Published:** 2011-02-10

**Authors:** Anna K. Jäger, Lasse Saaby

**Affiliations:** Department of Medicinal Chemistry, Faculty of Pharmaceutical Sciences, University of Copenhagen,2 Universitetsparken, 2100 Copenhagen, Denmark; E-Mail: las@farma.ku.dk (L.S.)

**Keywords:** flavonoids, CNS, mental health, GABA, MAO

## Abstract

Flavonoids are present in almost all terrestrial plants, where they provide UV-protection and colour. Flavonoids have a fused ring system consisting of an aromatic ring and a benzopyran ring with a phenyl substituent. The flavonoids can be divided into several classes depending on their structure. Flavonoids are present in food and medicinal plants and are thus consumed by humans. They are found in plants as glycosides. Before oral absorption, flavonoids undergo deglycosylation either by lactase phloridzin hydrolase or cytosolic β-glucocidase. The absorbed aglycone is then conjugated by methylation, sulphatation or glucuronidation. Both the aglycones and the conjugates can pass the blood-brain barrier. In the CNS several flavones bind to the benzodiazepine site on the GABA_A_-receptor resulting in sedation, anxiolytic or anti-convulsive effects. Flavonoids of several classes are inhibitors of monoamine oxidase A or B, thereby working as anti-depressants or to improve the conditions of Parkinson’s patients. Flavanols, flavanones and anthocyanidins have protective effects preventing inflammatory processes leading to nerve injury. Flavonoids seem capable of influencing health and mood.

## 1. Introduction

Throughout time, many plants have been used for the treatment of mental problems. A good number of those are alkaloid-containing plants, as alkaloids are known to interact strongly with receptors in the central nervous system, but in recent years it has become clear that flavonoids may also play a role in enzyme- and receptor systems of the brain, exerting various effects on the central nervous system, including prevention of the neurodegeneration associated with Alzheimer’s and Parkinson’s diseases. 

Plants where flavonoids are thought to, or are proven to be active constituents, include species with a long history of use as traditional folk medicines in Europe. Camomile flowers (*Matricaria recutica* L. — Asteraceae) have for centuries been used for their calming effect, which is due to apigenin [1]. A bioassay-guided fractionation lead to the isolation of apigenin as the constituent which caused GABA-benzodiazepine receptor activity from feverfew (*Tanacetum parthenium* L. — Asteraceae), which is used as a prophylactic remedy for treatment of migraine [2]. Linden flowers (*Tilia* sp. — Tiliaceae) have been used around the world as a tranquiliser, and it has been shown that quercetin- and kaempferol flavonoids are responsible for the sedative effect [3]. Heather, (*Calluna vulgaris* (L.) Hull. — Ericaceae) which traditionally has been used as a nerve calming remedy, was found to possess MAO-A inhibitory activity, with quercetin being the active component [4]. 

Flavonoids possess a variety of biological activities, besides their effects on the CNS. They have attracted attention as free radical scavengers with antioxidant activity. They are yellow, blue or red, and function as both UV-protection for the plant and pollination aids by providing specific colours or patterns to flowers. More than 6,000 flavonoids are known. 

**Figure 1:**
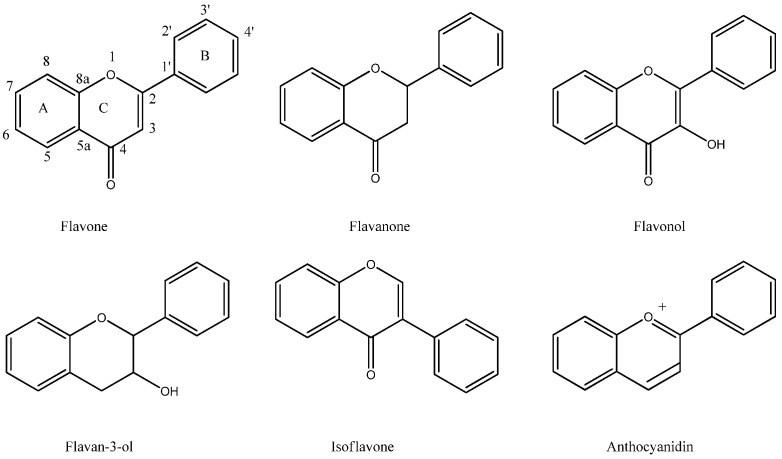
Structures of flavonoids.

Flavonoids can be divided into different classes depending on their oxidative status and substituents (Figure 1). They are biosynthesised via the acetate- and shikimic acid pathways, resulting in a C6-C3-C6 skeleton consisting of two aromatic rings and an oxygen-containing heterocyclic benzopyran ring. The labelling of the ring systems has the fused aromatic ring as A, the benzopyran ring adjacent to A as ring C, and the phenyl as ring B.

Flavonoids belonging to the flavones, flavonols and isoflavones groups have a double bond in ring C, which means that the fused A-C ring system is planar. The other flavonoids lacking a double bond in ring C have C2 and C3 placed on each side of the plane of the A ring. These flavonoids also have chiral centres at C2 and C3.

In Nature, flavonoids often occur as polymers, with dimers being the most common form. The flavonoids are linked through C-C or C-O-C bonds. The ubiquitous flavone apigenin is found as amentoflavone (3,8”-biapigenin), agathisflavone (6,8”-biapigenin), cupressiflavone (8,8”-biapigenin) formed via C-C linkages of two apigenin molecules, and as hinokiflavone (4’,6”-biapigenin) via C-O-C bonds [5]. The two monoflavonoid-units of the biflavone may or may not be of the same type. An example of a trimer is epigallocatechin, which consists of three C-C linked catechin molecules.

Flavonoids in food or medicinal plants must be absorbed in the digestive tract, and then transported by the circulatory system to the brain, where it must pass the blood-brain barrier before they can exert any effect on the CNS.

## 2. Oral Bioavailability of Flavonoids

Most flavonoids, apart from cathecins, are usually present in plants as β-glycosides. Prior to absorption into the systemic circulation these glycosides must undergo deglycosylation [6]. This process occurs predominantly in the intestinal lumen, mainly through the action of two enzymes: lactase phloridzin hydrolase (LPH) and cytosolic β-glucocidase (CBG). LPH is membrane bound on the luminal surface of enterocytes and aglycones liberated by this enzyme will therefore be released into the intestinal lumen where they are able to passively diffuse across the intestinal membrane [7,8]. CBG is capable of hydrolyzing a broad range of glycosides including glucosides, galactosides, xylosides, arabinosides, and fucosides [8,9]. However, as this enzyme is located intracellularly in the enterocytes, active transport of the hydrophilic glycosides into the cells via the sugar transporter SGLT-1 is required [8]. Glycosides that are not substrates of LPH or SGLT-1 will be transported into the colon where bacteria are able to hydrolyze the flavonoid glycosides [10]. However, due to bacterial degradation of the liberated aglycones and the reduced absorption capacity of the colon, only minor absorption of the flavonoid glycosides entering the colon can be expected [11]. Once absorbed the flavonoid aglycones are subjected to three main types of conjugation: methylation, sulphatation and glucuronidation [12]. The capacity for conjugation is high and the plasma concentration of free flavonoid aglycones is usually very low suggesting an extensive conjugation of absorbed flavonoid aglycones [12]. The presence of conjugated metabolites in the portal blood in rats suggests that conjugation of flavonoids first occurs in the enterocytes before further conjugation in the liver [13,14]. This could also be the case in humans, as the *in vitro* glucurunidation of quercetin and luteolin is markedly higher in human intestinal microsomes than the glucuronidation in human liver microsomes [15].

### 2.1. Flavan-3-ols

In a human study, ten healthy subjects were asked to consume 500 mL of green tea which contained 648 µmol of flavan-3-ols [mainly, (-)-epigallocatechin-3-*O*-gallate, (-)-epigallocatechin and (-)-epi-catechin], after which plasma and urine was collected over a period of 24 h [16]. Analysis of the collected plasma revealed a total of twelve metabolites in form of *O*-glucuronidated, methylated and sulphate conjugates of epicatechins and epigallocatechins and the unmetabolized flavan-3-ols (-)-epigallocatechin-3-*O*-gallate and (-)-epicatechin-*O*-gallate. Detectable quantities of the flavan-3-ols were present in the plasma 30 min after consumption of the green tea. The predominant metabolite detected in plasma was epigallocatechin-3-*O*-glucoronide, reaching a C_max _of 126 nmol/L and a T_max _of 2.2, while the lowest amount detected was of (-)-epicatechin-3-*O*-gallate with an C_max _of 25 nmol/L and a T_max _of 1.6 h. In another study human subjects were given a single dose of 20 mg/mL green tea solids dissolved in warm water [17]. Subsequent quantification of (-)-epigallocatechin-3-*O*-gallate, (-)-epigallocatechin and (-)-epicatechin revealed C_max_ values of 78 ng/mL, 223 and 124 ng/mL (total of the free aglycone and conjugates), respectively. The time to reach C_max _values was in the range of 1.3-1.6 h. Corresponding AUC values were 508.2, 945.4 and 529.5 ng h mL^-1^ respectively.

### 2.2. Flavonols

In a human feeding study, subjects were given an acute dose of 275 µmol flavonol glucosides in the form of fried onions with the main constituents being the 4’-*O*-glucoside and the 3,4’-*O*-glucoside [18]. Analysis of plasma revealed five predominant quercetin metabolites: quercetin-3’-*O*-sulfate, quercetin-3-*O*-glucuronide, isorhamnetin-3-*O*-glucuronide, quercetin-*O*-glucuronide*-O*-sulfate and quercetin-*O*-diglucuronide. The principal metabolites were quercetin-3’-*O*-sulfate (C_max_ of 665 nmol/L, T_max _0.8 h) and quercetin-3-*O*-glucuronide (C_max_ of 351 nmol/L, T_max _0.6 h). The lowest C_max _(62 nmol/L) was detected for quercetin-*O*-diglucuronide, with a T_max _of 0.8 h. The bioavailability of quercetin-3-*O*-rutinoside was studied in human subjects given a single dose of 176 µmol of the quercetin rhamnose-glucose disaccharide in the form of 250 mL tomato juice [19]. In this study only two metabolites were detected in plasma, namely quercetin-3-*O*-glucuronide and isorhamnetin-3-*O*-glucuronide with C_max _values of 12 nmol/L and 4.3 nmol/L, respectively. For both metabolites a T_max _value of around 5 h was observed. 

### 2.3. Flavanones

The bioavailability of the flavanones hesperetin-7-*O*-rutinoside and naringenin-7-*O*-rutinoside was investigated in human volunteers given 250 mL orange juice which corresponded to a dose of 168 µmol and 12 µmol of the two flavanones, respectively [20]. Two metabolites were detected in the plasma: hesperetin-7-*O*-glucuronide and a hesperetin-*O*-glucuronide, which was not further assigned. The total C_max _value of the two metabolites was 922 nmol/L with a T_max _ca. 4 h. In a similar study where the bioavailability of hesperetin and naringenin was investigated in human subjects given a dose of 444 mg hesperetin and 96 mg narirutin in 1 L orange juice [21], peak plasma concentration of hesperitin metabolites was 1.3 µmol/L and 0.2 µmol/L for naringenin metabolites. Concentration of flavanone metabolites peaked between 5 and 7 h. Distribution of hesperetin metabolites was glucuronides (87%) and sulphoglucuronides (13%). Due to the low plasma concentration it was not possible to determine the distribution of naringenin metabolites. 

### 2.4. Isoflavones

In a human study investigating the bioavailability of the pure isoflavones genistein and daidzein, volunteers was given a dose of 0.8 mg/kg administered in gelatin capsules [22]. Subsequent analysis of plasma samples revealed peak concentrations (total of all metabolites) of 0.7 µmol/L for genistein metabolites and 0.9 µmol/L for daidzein metabolites. Time to reach peak concentration was about 7 h. In another human study the bioavailability of genistein-7-glucoside and daidzein-7-glucoside was investigated in volunteers receiving tablets containing 62 µmol and 55 µmol of the two pure isoflavone glucosides, respectively [23]. Plama analysis revealed peak plasma concentrations of 0.4 µmol/L for genistein metabolites and 0.2 µmol/L for daidzein metabolites. Peak plasma concentrations were reached in the range of 4-6 h. 

### 2.5. Anthocyanidins

In a human study, subjects was given 200 g of strawberries containing 222 µmol pelargonidin-3-*O*-glucoside and small amounts of pelargonidin-3-*O*-rutinoside (13 µmol) and cyanidin-3-*O*-glucoside (6 µmol) [24]. Plasma samples contained one major metabolite, pelargorinidin-*O*-glucoronide, and three other pelargorinidin-glucoronides and a pelargorinidin-rutinoside in amounts that was not quantifiable. The peak plasma concentration of pelargorinidin-*O*-glucoronide was 274 µmol/L at a T_max _of about 1 h. 

## 3. Flavonoid Permeability across the Blood-Brain Barrier

The blood-brain barrier (BBB) is mainly formed by brain capillary endothelial cells, however other cell types such as pericytes, astrocytes and neuronal cells also play an important role [25]. Brain capillary endothelial cells differ from peripheral endothelial cells as brain endothelial cells have tight junctions which prevent paracellular transport of small and large water-soluble compounds from blood to the brain [25]. Transcellular transport is further limited due to low vesicular transport and high metabolic activity. Altogether, the BBB functions as a physical and metabolic barrier [25]. A prerequisite for CNS activity is therefore that flavonoids and their conjugates are able to traverse the BBB and enter the CNS. 

In an *in vitro* study two aspects of the interaction between flavonoid molecules with the BBB was examined: the uptake of selected flavonoids, and their glucuronides into two brain endothelial cell lines (b.END5 and RBE4) and their permeability across an *in vitro* model of the BBB (ECV304 cells co-cultured with C6 glioma cells) [26]. The two brain endothelial cell lines were treated with 30 µM solutions of hesperetin, naringenin, epicatechin and their glucuronides. Cyanidin-3-rutinoside and pelargonidin-3-glucoside was also included in the study. Flavonoids showing the highest degree of uptake into b.END5 and RBE4 cells were hesperetin (140 and 146 ng/mg protein, respectively) and naringenin (177 and 127 ng/mg protein, respectively). The uptake into b.END5 and RBE4 cells was markedly lower for hesperetin glucuronide (6 and 7 ng/mg protein, respectively), naringenin glucuronide (7 and 9 ng/mg protein, respectively), cyanidin-3-rutinoside (26 and 19 ng/mg protein, respectively) and pelargonidin-3-glucoside (18 and 11 ng/mg protein). Uptake of epicatechin and its glucuronide could not be detected. An interesting finding was that the glucuronides of hesperetin and naringenin were metabolized to the parent aglycone upon uptake into b.END5 and RBE4 cells. The above observations were reflected in the permeability of the flavonoids across a monolayer of ECV304 and C6 glioma cells. The order of permeability was naringenin > hesperetin > naringenin glucuronide > hesperetin glucuronide > cyaniding-3-rutinoside > pelargonidin-3-glucoside. Flux across the monolayer could not be detected for epicatechin and its glucuronide. 

In an *in vivo* study rats were given a standardized *Ginkgo biloba* extract (EGb761®) containing 22-27% flavonoid glycosides, mainly glycosides of quercetin, kaempferol and isorhamnetin [27]. After a single dose of 600 mg/kg of EGb761 only kaempferol and isorhamnetin could be detected in the rat brain tissue with maximum concentrations of 293 and 161 ng/g protein (total of aglycone and conjugates), respectively. Although quercetin was not detected in whole brain homogenates, relatively high concentrations was detected when the hippocampus, stratum and cerebellum were analyzed separately (>1,000 ng/g protein). Corresponding total aglycone and conjugate amounts of kaempferol and isorhamnetin were around 1,000 and 2,000 ng/g protein, respectively. 

The content of flavoniods in brain tissue is given per mass of total protein analyzed (e.g., ng/g). This value cannot easily be used for estimates of intracellular molar concentrations of flavonoids, making it difficult to compare values with results obtained from *in vitro* studies.

## 4. *In Vivo* Effects

Flavonoids have been investigated in *in vivo* models, where effects on the CNS have been demonstrated. The flavonoid glycosides linarin, 2*S*-hesperidin, 2*S*-neohesperidin, 2*S*-naringenin, diosmin, gossipyn and rutin were found to exert a depressant action on the CNS of mice following i.p. injection, measured in the hole board, thiopental induced sleeping time and locomotor activity tests [28]. The aglycones were inactive, pointing to the importance of the sugar moieties in the glycosides for CNS activity. These results are surprising, but it should be kept in mind that the compounds were given i.p. and thus absorption and metabolism were altered compared to oral administration of the flavonoids. The aglycone apigenin showed no anticonvulsant or anxiolytic properties *in vivo* [1,29]. On the other hand, hispidulin, which is 6-methoxyapigenin, had anticonvulsive activity in a model of epilepsy in seizure-prone Mongolian gerbils [30]. 

Chrysin, a flavone from *Passiflora coerulea* L.(Passifloraceae) increased the number of entries into and time mice spent in the open arms in the elevated plus-maze test of anxiety, consistent with an anxiolytic action. Chrysin also increased the time spent on head-dipping. In contrast, chrysin had no myorelaxant action, indicating that chrysin possesses anxiolytic [31]. The flavone ororylin A isolated from the Chinese medicinal plant *Scutellaria baicalensis* Georgi (Lamiaceae), which is used for sedation, showed selective sedative and anticonvulsant activity *in vivo*, which support a subtype specific activity on the GABA_A_-receptor of the flavone [32]. Epigallocatechin, a trimer of catechin which is found in green tea, had dose-dependent anxiolytic, sedative and amnesiac activity, likely mediated via the GABA_A_-receptor [33,34].

The *in vivo* studies show that flavonoids are able to be absorbed after oral administration, pass the blood-brain barrier and do have various effects on the CNS.

## 5. GABA_A_-benzodiazepine Receptor

### 5.1. The GABA_A_ receptor

GABA is the most important inhibitory neurotransmitter in the human central nervous system. GABA is involved in epilepsy, sedation and anxiolysis, and works via binding to GABA_A_ receptors. GABA_A_ receptors are heteromeric GABA-gated chloride channels. The transmembrane ion channel is opened by a stimulus generated by GABA, which allows an influx of chloride ions. This results in a decrease of the depolarizing effects of an excitatory input, thereby depressing excitability [35]. As a result the cell is inhibited and an anticonvulsant, sedative or anxiolytic activity is achieved. The type of activity obtained depends on the subtype of the receptor. The GABA_A_ receptor consists of five subunits, made up of two α, two β and one γ or δ subunit. Several isoforms exists (α1-α6, β1-β3, γ1-γ3, δ), potentially giving a vast number of combinatorial mixes. However, only ten subunit combinations make up the physiologically relevant GABA_A_ receptors in the brain [36,37].

Besides the binding site for the neurotransmitter itself, there are modulatory binding sites on the receptor. Benzodiazepines bind to the so-called benzodiazepine site, where they modulate the receptor to be more sensitive to GABA, and thereby yielding an anticonvulsant, sedative or anxiolytic effect. 

### 5.2. Flavones

A number of monoflavonoids with affinity to the GABA_A_-benzodiazepine site have been isolated from plants. Apigenin was isolated due to the compound’s affinity to the benzodiazepine site from *Matricaria retutica* (Asteraceae) [1], *Tanacetum parthenium* (Asteraceae) [2] and *Sersia dentata* Thunb. (Anacardiaceae) [38]. A number of K_i-_values have been reported: 3 μM [39,40], 4 μM [1], 9 μM [2] and 40 μM [38]. 6-methylapigenin was isolated from the sedative plant *Valeriana wallichii* D.C. (Valerianaceae), and had a Ki-value 495 nM [41]. Dinatin, skrofulein, cirsilineol and hispidulin were isolated from *Artemisia herba-alba* Asso (Asteraceae), and IC_50_-values of 1.3 µM, 23 µM, 104 µM and 8 µM were reported, respectively [42,43]. Chrysin was isolated as the active compound in *Passiflora coerulea* (Passifloraceae) with a Ki-value of 3 µM [44].

The active monoflavononoids isolated from plants are all flavones. The flavanone (*S*)-naringenin, isolated in a bioassay-guided isolation from *Menthaaquatica* L.(Lamiaceae), had a high IC_50_-value of 2.6 mM [45], and thus has very low activity. This indicates that the double bond in the C-ring is important for activity, yielding a planar structure for the A-C ring system.

### 5.3. Biflavones

The biflavonoid amentoflavone is found in a number of plants with medicinal properties, including *Ginkgo biloba* L. (Ginkgoaceae)*,Hypericum perforatum* L. (Clusiaceae) and *Searsia pyroides* Burch. (Anacardiaceae)*.* It has been shown to exhibit high affinity to brain benzodiazepine receptors *in vitro* [46], with K_i_ values of 6 nM [47], 7 nM [39,40] and 56 nM [38]. Studies on subtype-specificity showed that amentoflavone had little or no affinity for α4- or α6-containing receptors [48]. The biflavonoid agathisflavone isolated from *Sersia pyroides* inhibited ^3^H-flumazenil binding with a K_i_ of 82 nM [38]. Further functional characterization of the *Searsia pyroides* extract, as well as apigenin, amentoflavone and agathisflavone, showed inhibitory effects on spontaneous epileptiform discharges in mouse cortical slices of the plant extract, but no effects of the isolated flavonoids, indicating that the anticonvulsant effect is due to involvement of a different neurotransmitter system [49]. 

### 5.4. Two binding sites for flavonoids

Interestingly, there might be two binding sites on the GABA_A_ receptor where flavonoids bind. Electrophysiological studies have indicated that there might be a high-affinity flumazenil-sensitive site, and another low-affinity, flumazenil-insensitive site. Further, it has been shown that apigenin, hesperidin and (-)-epigallocatechin gallate enhance the activity of the clinically used benzodiazepine diazepam [50,51], by a modulatory action not fully understood.

### 5.5. Subtype specificity

Some flavonoids have been shown to have subtype specific activity. It is reported that 6-hydroxyflavone acted as a subtype selective partial positive allosteric modulator at the flumazenil sensitive benzodiazepine site. 6-hydroxyflavone had stronger affinity to receptors containing α2 and α3-subunits, than to receptors with α1 and α5-subunit. *In vivo*, 6-hydroxyflavone exhibited anxiolytic effects, without sedation, cognition impairment, motor incoordination or convulsions [52]. Further investigations of subtype specificity of flavonoids might lead to identification of flavonoids with selective pharmacological activities, thus providing a clinically interesting lead structure.

## 6. Monoamine Oxidase

Monoamine oxidase (MAO) is a flavoenzyme found in the outer membrane of mitochondria. MAO catalyzes the oxidative deamination of primary, secondary and some tertiary amines [53]. Two isoforms of MAO exists: MAO-A and MAO-B, where MAO-A preferentially oxidizes serotonin (5-hydroxytryptamine) and noradrenaline, whereas MAO-B preferentially oxidizes phenylethylamine [54]. Dopamine and tyramine appears to be substrates for both isoenzymes. In the CNS, MAO-A is present in the extraneuronal compartment and within the dopaminergic, serotonergic and noradrenergic nerve terminals, while MAO-B is mainly localized in the glial cells [55]. The primary roles of MAO-A and MAO-B lie in the metabolism of exogenous amines and in the regulation of neurotransmitter levels and intracellular amine stores [54]. It is believed that the pathology of depression involves a deficiency of 5-hydroxytryptamine and noradrenaline, and selective inhibitors of MAO-A are therefore used in the treatment of depression. Selective MAO-B inhibitors are used in the treatment of Parkinson’s disease. Treatment of Parkinson’s disease aims at compensating for the deficit in dopaminergic activity and because dopamine is preferentially deaminated by MAO-B, inhibition of this isoenzyme should raise the basal central level of dopamine [56].

### Interactions between flavonoids and monoamine oxidase

Several flavonoids have been identified as inhibitors of MAO-A and MAO-B. The flavonols kaempferol and quercetin and the flavones apigenin and chrysin were isolated from a standardized *Gingko biloba* extract by means of HPLC [57]. All four flavonoids were identified as MAO-A inhibitors with IC_50 _values of: kaempferol (0.7 µM), apigenin (1 µM), chrysin (2µM) and quercetin (5 µM). Phenelzine, a non-selective and irreversible inhibitor of MAO was used as a reference compound (IC_50_ value 0.04 µM). Quercetin was isolated from heather (*Calluna vulgaris* (L.) Hull – Ericaceae) and identified as a MAO-A inhibitor with an IC_50 _value of 18 µM. In the same assay clorgylin, a selective MAO-A inhibitor, had an IC_50 _value of 0.2 µM [4]. In another study it was reported that quercetin is a selective MAO-A inhibitor with an IC_50_ value of 0.01 µM for MAO-A and 20 µM for MAO-B [58]. Quercetrin, isoquercetrin, rutin and quercetin isolated from *Melastoma candidum* D. Don (Melastomataceae) were shown to inhibit MAO-B with IC_50_ values of 19, 12, 4, 11 µM, respectively, in an assay where deprenyl (a selective MAO-B inhibitor) had an IC_50_ value of 19 µM [59]. The flavan-3-ols (+)-catechin and (-)-epicatechin were isolated from *Uncaria rhynchophylla* (Miq.) Jacks. (Rubiaceae) and found to inhibit MAO-B with IC_50 _values of 89 and 59 µM, respectively, while deprenyl had an IC_50_ value of 0.3 µM [60]. Two flavonoids isolated from *Sophora flavescens* Ait. (Fabaceae) exhibited monoamine oxidase inhibitory activity: formononetin an isoflavone with IC_50_ values of 21 µM (MAO-A) and 11 µM for MAO-B and the flavanone kushenol F with IC_50_ values of 104 µM (MAO-A) and 63 µM for MAO-B [61]. Naringenin was isolated from *Mentha aquatica* L. (Lamiaceae) in a bioassay-guided fractionation process [62]. The IC_50_ value for MAO-A inhibition was 955 µM and 288 µM for MAO-B in an assay where the IC_50 _value of clorgylin was 0.0003 µM and 0.1 µM for deprenyl. In a recent study the inhibitory effects of pure anthocyanidins on MAO-A and MAO-B activity was investsigated [63]. The following IC_50_ values were obtained for MAO-A and MAO-B inhibitory activity, respectively: malvidin (22 µM and 19 µM), pelargonidin (27 µM and 43 µM), cyanidin (30 µM and 32 µM), peonidin (31 µM and 22 µM), petunidin (32 µM and 43 µM), delphinidin (35 µM and 31 µM). In the same study different glycosides and diglycosides of the above mentioned anthocyanidins were also studied with IC_50_ values in the range of 29-117 µM for MAO-A inhibition and 31-242 µM for MAO-B inhibition. 

All of the active flavonoids identified in the above possess inhibitory activity on MAO-A, MAO-B or both. Furthermore, this inhibitory activity is not confined to a single flavonoid class as all the classes presented in figure 1 are represented. 

## 7. Cognition and Neurodegeneration

Evidence is mounting that flavonoids have effect on memory, cognition and neurodegeneration. Although many of the studies in this field have been performed with flavonoid-rich fruits, both these studies and studies with isolated flavonoids indicate that flavonoids have potential to protect neurons against injury induced by neurotoxins and neuroinflammation, a potential to activate synaptic signalling and an ability to improve cerebrovascular blood flow, as has recently been reviewed [64].

Studies with fruit supplements indicate that flavanols, flavanones and anthocyanidins have the capacity to improve memory. (-)-Epicatechin has been shown to improve rat spatial memory in the water maze test [65]. It is thought that the flavonoids exert their action via influencing signalling pathways involved in the normal memory processing, but the precise mechanism of action has not been elucidated. Flavonoids are known to affect endothelial function and peripheral blood flow [5,66]. In the same way, they may be beneficial in prevention of cerebrovascular problems, however the effect of pure flavonoids is not well investigated. Several flavonoids have been shown to protect against neuronal injury. Epicatechin, 3’-*O*-methyl-epicatechin and hesperetin protected neurons against oxidative neuronal damage [67,68]. The flavanone naringenin was able to inhibit inflammatory processes leading to neuronal cell injury [69].The flavonol quercetin and the flavan-3-ols catechin and epigallocatechin gallate also affect neuroinflammation [70,71]. 

## 8. Conclusions

Dietary flavonoids are absorbed as aglycones from the intestinal lumen and subsequently conjugated in the enterocytes to glucuronides, sulphates and methylates of the parent aglycone. Thus, flavonoids are present in the plasma almost exclusively in a conjugated form. Flavonoids, both as aglycones and in the conjugated form pass the blood-brain barrier. During passage of the blood-brain barrier conjugates may be metabolized back to the parent aglycone, which then enters the central nervous system. In most studies the compounds isolated from the plant e.g., aglycones and glycosides, were tested, where as the effect of conjugation are rarely considered. In the brain flavanoids act on different systems. Flavanols, flavanones and anthocyanins may act in protective ways, increasing the cerebral blood flow and protecting the neurons against inflammatory processes leading to cell injury. Flavones may interact with the GABA_A_-receptor, producing sedation, anxiolytic or anticonvulsive effects. Flavonoids of several classes are inhibitors of MAO-A and -B, thus mediating an anti-depressant or anti-Parkinson’s activity. 

With this background it seems plausible that the diet may influence a person’s health state and even mood. Plants that are rich in flavonoids of the flavanol, flavanone and anthocyanin-type are berries, citrus fruits, apple, pear, tea, cocoa and wine, which are common food products. It should be noted that the plants rich in GABA-active flavones are not common food plants, but fall within the category of medicinal plants. It thus seems that over time humanity have found plant products suitable for consumption as functional foods, and others that are left for medicinal purposes.
